# German

**Published:** 1897-04

**Authors:** J. Preston Hoskins

**Affiliations:** Princeton, N. J.


### ABSTRACTS FROM FOREIGN JOURNALS.

### GERMAN.

UNDER THE DIRECTION OF J. PRESTON HOSKINS,
PRINCETON, N. J.

PECTORAL FORM OF SEPTICÆMIA HEMORRHAGICA. According to the investigation of Van Eecke, in the last six months a contagious cattle-disease broke out in the districts of Bintenzorg and Mt. Connelis, which caused many deaths. The government veterinarian Fischer published a detailed report of the same which appeared in the *Veeartsenijkundige Bladen.*

The disease assumed here and there a malignant character. In the district of Tjibaroesa, where the disease seems to have first appeared, of about 500 buffaloes 100 died on a single ranch within a short time (September to October), while on many other ranches the death-rate reached 10 per cent. The total number of deaths in the department mentioned was estimated at upward of 700 out of a total of 14,000 buffaloes. From the nature of the case, such an estimate is too low. The interests of the cattle-owners demanded that the existence of the disease be kept secret. Many of the infected animals were slain in the early stages in order to place them on the market. Herr Fischer, who made the post-mortem examination of the first case, makes the following report: The animals lay apathetic on the ground and refused food and drink; wounds of any kind were not observed. The nutrition of the young animals was very good; breathing was rapid and labored; the pulse was increased and weak. The temperature of the body was to the touch considerably elevated. The nostrils were dry; the eyes appeared sunken, and the conjunctivæ were colored red; he noticed tears flowing from the eyes. With the consent of the owner, the animal was killed that a post-mortem might be held. On account of the good condition of the animal only a little dark blood flowed off. When the skin was removed minute hemorrhages presented over the whole body, being especially prominent in the muscles, which were dry to the touch. When the abdominal cavity was opened nothing abnormal in the position of the intestines was noticed. The injection of the bloodvessels was especially marked. The thoracic cavity, on the other hand, showed important changes. On opening the cavity about one litre of clear yellow serous fluid flowed out. This quantity was considerably increased when a sero-fibrous layer, 2 to 3 cm. thick, was removed from the rib-wall. This layer formed an inner connection between the rib-wall and the lung, and covered all parts of the pleura to a greater or less extent, except the diaphragm, which with the exception of red spots and stripes upon it was normal. The sero-fibrous layer, which was at first 5 cm. thick, decreased in consequence of the escape of fluid to 1 mm. The lungs, after the layer was removed, were found spotted and striped dark red. This was most noticeable on the front half. They were not shrunken, but very friable and tore easily, a serous bloody fluid flowing off. On the inside the lung presented a marbled appearance which was caused chiefly by obtrusion of the interstitial tissues. Light yellow streaks were found through the whole lung, the thickness of which varied between 2 mm. and 1½ cm. On pressure liquid exuded from them and the thickness was reduced to 1 mm. or less. The tracheal and bronchial mucous membrane was dark red over the whole surface and covered with a foul-looking layer of slime. The mucous membrane of the mouth and nose was somewhat injected with blood, that of the throat a dark color. The pericardium contained a small quantity of liquid, while the peri-, epi-, and endocardium showed dark-red spots. The wall of the heart was flabby and the cavities filled with large white coagulations which were floating in the dark-colored blood. The coagulations extended to the large cavities. The pouch and stomach were filled with free, hard food-stuff. The mucous membrane of the intestines was somewhat hyperæmic, the contents thin. The capsule of the spleen showed some spots. The spleen was not enlarged. The liver was slightly spotted, as were also the kidneys, but otherwise the latter were normal. The chylous glands of the intestines were somewhat enlarged and exuded liquid when cut. The bladder was normal. The provisional diagnosis was a pectoral form of hemorrhagic septicæmia. In the course of the contagion more than twenty post-mortems were held. In all were found to a greater or less extent the deviations in the thoracic cavity mentioned above. When these were slight, changes in the abdominal cavity were marked. Here hyperæmia and swelling of the mucous membrane of the whole digestive tract were present. In places a peeling off of the epithelium was noticeable, but without ulcers and not affecting the follicular apparatus. The first three stomachs were filled with hard, dry food, and the intestines with thin pappy matter. The peritoneum had a spotted appearance, yet even where the abdominal cavity was most severely affected the color of the intestines was such as to exclude all possibility of splenic fever. The liver was normal in size and dark-colored, spotted, and very brittle. The gall-bladder was spotted dark-red and filled with dark gall. The spleen was not enlarged, of normal consistency, with the capsule spotted dark-red. The kidneys, of normal size, were dark-red with black spots here and there. The mucous membrane of the genito-urinary apparatus was spotted and striped with red. The urine was not bloody. Besides the above-mentioned, other forms of the disease appeared which were given various names by the people. Fischer saw only a couple of these cases. They were marked by a severe œdema of the subcuticle which was present on the neck, over the breast and belly and a portion of the limbs. These appearances agree with those of the cutaneous form of Bollinger's cattle epidemic, which was the subject of a prior investigation by Van Eecke. The appearance and disappearance of the œdema contemporaneously with the pectoral and abdominal form, as well as the striking likeness in the conditions on post-mortem examination, caused Fischer to incline to the opinion that they are connected etiologically with each other and must be considered as septicæmia hemorrhagica. As soon as a favorable opportunity offered to send some fresh material to the laboratory for investigation, use was made of it by the officials in charge. The material received was examined by Van Eecke, from whose results we take the following: The case under consideration was one in which a diagnosis of pectoral form of septicæmia hemorrhagica had been made. The material for investigation reached the laboratory a couple of hours after the post-mortem, and consisted of blood from the large vessels and a piece of the sero-fibrinous mass, described above, from the thoracic cavity. A very small quantity of the blood was injected in the skin of a rabbit. The following day the animal was found dead. On examination the condition was the same as Van Eecke has described. Although it was a case of the pectoral form of the disease, no important changes in the pectoral organs could be demonstrated. From the blood of the original buffalo, as well as from the blood in the organs of the rabbit, microbes of septicæmia hemorrhagica were cultivated and their identity established by continued culture- and infection-tests. Through lack of rabbits no further tests could be made with these animals. Later, however, some of these animals fell a victim to this disease in consequence of accidental infection. They were probably infected by the excrement of artificially-infected doves whose cages hung above the rabbit warren. On post-mortem examination the following conditions were noted: subcutaneous œdema in the submaxillary and neck region (but only in two of the four animals), combined with hemorrhages in the region of the shoulder and groin. A moderate quantity of clear, serous fluid was found in the peritoneal and pleural cavities, and a considerable quantity of the same fluid in the pericardial cavity. The heart had stopped beating in diastole. Hemorrhage and œdema were present in the lungs, which sunk in water. Tracheitis hemorrhagica present. The spleen of normal consistency, but somewhat enlarged; hyperæmia of the liver; swelling and œdema in the kidneys; hyperæmia in the mucous membrane of the œsophagus, stomach, and duodenum. The small intestines contained a light yellow, slimy matter. The rectum was empty, or contained thick, greenish-brown feces. The retroperitoneal glands swollen and sappy. The bladder distended with muddy urine. In the blood of all the animals the presence of septicæmia hemorrhagica microbes was demonstrated not only microscopically, but also by culture- and infection-tests. The infection-tests were made especially on turtle-doves (*Turtur tigrimis* and *bitorquatus*), animals of little value and easily procured, which react in a typical manner after intra-muscular injection of the virus. The serous fluid from the sero-fibrinous mass taken from the pectoral cavity of a buffalo was also injected into a turtle-dove, with positive result. Under the microscope this fluid seemed to contain the bacteria which cause the typical discoloration, in great mass. These were also cultivated and other animals infected with the cultures with positive result. In some cases pectoral changes were found after death, but in the majority of cases none. When material from a dove that had died of the pectoral form was injected into another dove the latter almost always showed the pure septicæmic form. Even in the earlier tests also, where the original infectious matter was taken not from the pectoral but from the cutaneous form of the disease, Van Eecke found in single cases some sharply-defined bloody spots in the lungs which otherwise showed no deviation from those of healthy doves.

Tests were also made on mice, rats, sparrows, and a pair of goats. The mice died in from one to two days after the injection. In the case of the rats the result was less satisfactory Some were infected by subcutaneous injections, others through the food. They died after one to three days, and showed on examination the phenomenon of pure septicæmia hemorrhagica. But the investigator did not succeed in finding the specific microbes. The sparrow died within twenty-four hours after an intra-muscular injection of 0.15 cm. of the bouillon-culture. It was followed by marked change in both lungs and the hydropericardium; many septicæmia hemorrhagica bacilli were found in the blood with the microscope. One of the goats was infected subcutaneously with an agar-culture divided in bouillon from the serum of a buffalo; the other with the blood of one of the doves which had died from the disease. Both showed only a slight swelling at the place of injection. As bases for cultures alkali pepton bouillon and with it "glycerin-agar" were used mostly. Of importance, with a view to sending out plates of material for investigation from the laboratory, is the fact that from the blood and the serous fluid of the buffalo which is preserved at a temperature of 15°–20°, virulent septicæmia hemorrhagica microbes can be obtained after a week. From these investigations of Fischer and Van Eecke, the assertion of the latter, that much of what is generally regarded here in the country as cattle-pest is in fact nothing but septicæmia hemorrhagica, has not yet been fully proven. What Duessen wrote about the contemporaneous appearance of œdematous, gastric, and pneumonic forms of the cattle-pest should be compared with Fischer's report, and one will come to the conclusion that both have had to do with the same disease.—*Veeartsenijkundige Bladen voor mederlansch-indie*, vol. ix. No. 4.

The *Clinica Veterinaria*, of Milan, records the diagnosis of fracture of the sesamoid bones with the Röntgen rays.

